# Individuals with numerical and structural variations of sex chromosomes: interdisciplinary management with focus on fertility potential

**DOI:** 10.3389/fendo.2023.1160884

**Published:** 2023-05-05

**Authors:** Anders Juul, Claus H. Gravholt, Michel De Vos, Ekaterina Koledova, Martine Cools

**Affiliations:** ^1^ Department of Growth and Reproduction, Copenhagen University Hospital-Rigshospitalet, Copenhagen, Denmark; ^2^ Department of Clinical Medicine, University of Copenhagen, Copenhagen, Denmark; ^3^ Department of Endocrinology, Aarhus University Hospital, Aarhus, Denmark; ^4^ Institute for Clinical Medicine, Aarhus University, Aarhus, Denmark; ^5^ Department of Molecular Medicine, Aarhus University Hospital, Aarhus, Denmark; ^6^ Brussels IVF, Universitair Ziekenhuis Brussel, Vrije Universiteit Brussel, Brussels, Belgium; ^7^ Global Medical Affairs Cardiometabolic and Endocrinology, Merck Healthcare KGaA, Darmstadt, Germany; ^8^ Department of Internal Medicine and Pediatrics, Ghent University, Ghent, Belgium; ^9^ Pediatric Endocrinology Service, Department of Pediatrics, Ghent University Hospital, Ghent, Belgium

**Keywords:** DSD, Klinefelter syndrome, NSVSC, sex chromosomes, Turner syndrome

## Abstract

Diagnosis and management of individuals who have differences of sex development (DSD) due to numerical or structural variations of sex chromosomes (NSVSC) remains challenging. Girls who have Turner syndrome (45X) may present with varying phenotypic features, from classical/severe to minor, and some remain undiagnosed. Boys and girls who have 45,X/46,XY chromosomal mosaicism may have Turner syndrome-like features and short stature; therefore, unexplained short stature during childhood requires karyotype analysis in both sexes, particularly if characteristic features or atypical genitalia are present. Many individuals with Klinefelter syndrome (47XXY) remain undiagnosed or are only diagnosed as adults due to fertility problems. Newborn screening by heel prick tests could potentially identify sex chromosome variations but would have ethical and financial implications, and in-depth cost-benefit analyses are needed before nationwide screening can be introduced. Most individuals who have NSVSC have lifelong co-morbidities and healthcare should be holistic, personalized and centralized, with a focus on information, psychosocial support and shared decision-making. Fertility potential should be assessed individually and discussed at an appropriate age. Oocyte or ovarian tissue cryopreservation is possible in some women who have Turner syndrome and live births have been reported following assisted reproductive technology (ART). Testicular sperm cell extraction (TESE) is possible in some men who have 45,X/46,XY mosaicism, but there is no established protocol and no reported fathering of children. Some men with Klinefelter syndrome can now father a child following TESE and ART, with multiple reports of healthy live births. Children who have NSVSC, their parents and DSD team members need to address possibilities and ethical questions relating to potential fertility preservation, with guidelines and international studies still needed.

## 1 Introduction

Differences of sex development (DSD) include a diverse group of congenital conditions characterized by atypical development of chromosomal, gonadal, or phenotypic sexual characteristics. The diagnosis and management of DSD is frequently challenging, although understanding of the conditions has advanced rapidly in the last few years. While international guidelines for specific subgroups of DSD have been published (1, 2), there remains a need for further consensus. Knowledge gaps may lead to under-reporting of specific aspects of these conditions (3, 4).

Within the DSD classification, individuals who have numerical or structural variations of the sex chromosomes (NSVSC) represent a distinct group that has been associated with increased morbidity and mortality (5–7). Molecular karyotyping can identify the specific chromosomal constitution and will help with better classification of syndromic forms and provide information on co-morbidities, future fertility potential, and potential risk for the development of gonadal germ cell cancer (4, 5, 8, 9). National and international registries have been established for these and other DSD conditions, which will help to gain knowledge and understanding of the different conditions from real-world data, to provide structured follow-up and establish a sound basis for research (10–13).

Among individuals who have NSVSC, the most common are females with Turner syndrome, mainly non-mosaic 45,X monosomy, and males with Klinefelter syndrome, mainly non-mosaic 47,XXY trisomy. Pubertal delay, primary gonadal failure and reduced (Turner syndrome) or increased (Klinefelter syndrome) childhood growth occur frequently in these conditions. Various mosaic forms occur more rarely, including boys and girls who have 45,X/46,XY mosaicism. These conditions may or may not be associated with clinical features, including growth problems, atypical genital development and gonadal insufficiency.

## 2 Screening and diagnosis for patients with numerical or structural variations of the sex chromosomes

### 2.1 Diagnostic work-up

The birth of a baby who has a suspected NSVSC is often a challenging situation for both the parents and healthcare providers, especially in cases where no prenatal investigations were performed. Standardization of the diagnostic work-up across healthcare systems and clinical centers (13), is necessary as it enables evaluation of long-term consequences and optimized care options (4). It requires assessment of the baby by an interdisciplinary team of specialists, with accurate functional evaluation of several organ systems and a sensitive approach acknowledging that a DSD diagnosis can be challenging for patient and parents. Extensive counseling of the parents and providing effective psychosocial support are an essential component of this process (4, 14). In the presence of atypical genitalia, genital assessment should be detailed and standardized. The external genitalia score is a dedicated clinical tool covering the full spectrum of naturally occurring genital phenotypes. It has been developed and validated for genital assessment of premature and term babies up to the age of two years, including reference data for premature and low-birth weight babies (15). A sensitive approach can avoid psychological trauma caused by intrusive or otherwise inadequate genital assessment (16, 17).

Molecular karyotyping, complemented with comprehensive hormonal assessment, can determine the nature of the NSVSC (18, 19). Additional genetic investigations may be required to detect complex chromosomal rearrangements, low-grade mosaicism or mosaicism confined to specific tissues (20). The role of a dedicated clinical geneticist in translating the complex genetic information to the child and family requires special attention; an in-depth understanding of the genetics and subsequent impaired hormonal secretion, and how this may influence body development and gender identity, is essential for specialist healthcare providers, and remains among the most difficult challenges for patients and their families (20–23). In addition, accurate identification of the underlying genetic constitution enables improved counseling and future management of the condition, including planning pregnancies and identifying risks of transmitting genetic traits (3).

### 2.2 Turner syndrome

Girls who have Turner syndrome may present with a variety of karyotypes (**Table 1**) (24). Many girls with Turner syndrome have only minor phenotypic features and it is now recognized that there are few specific physical characteristics that can lead to an early diagnosis (1). Therefore, the diagnosis of Turner syndrome is often delayed, and may result in short final height if left untreated (25). Growth failure occurs in more than 95% and leads to diagnosis in many childhood cases (26). It is recommended to perform karyotyping in all females who are investigated for unexplained short stature or pubertal delay, whether or not in the presence of other characteristics such as lymphedema, micrognathia, left-side cardiac anomalies, hypertension or chronic ear infection (1, 18).

**Table 1:** Frequency of chromosomal anomalies in female patients with Turner syndrome.

Karyotype	Frequency
**45,X**	37%
**45,X/46,XX**	25%
**45,X/46,iso(X) and equivalents**	10%
**45,X/46,XY and equivalents**	4%
**Other karyotypes (including deletions, inversions and ring-formation)**	24%

Reproduced with permission from Gravholt et al., 2023, Endocr Rev (24).

A Danish study estimated the population-based prevalence of Turner syndrome as approximately 50 to 59 per 100,000 female births, which has gradually increased (5, 27). Interestingly, from 1961 to 2014, the incidence of 45,X Turner syndrome decreased, whereas other karyotypes associated with Turner syndrome increased (5). This has been attributed to increased detection of 45,X when a free prenatal screening program for Down’s syndrome was instituted in Denmark, leading to induced abortion in a number of cases. At the same time, fertility treatments became more widely available, leading to milder forms of Turner syndrome being detected due to fertility problems (28).

While there is a lack of nationwide information from many countries, with few data from America, Asia or Africa, registry studies in Denmark indicate that only approximately 70% of cases are identified (27). Also, extrapolation of data from Sweden and the UK indicated that only 60% and 34% of cases, respectively, were identified (5). Thus, the available data indicate that the current methods for diagnosing Turner syndrome are not sufficiently effective, and a case can be made for screening of entire populations. Neonatal screening programs could be adjusted to include accurate diagnosis of Turner syndrome, as well as extension of non-invasive prenatal testing with reporting of numerical variations of sex chromosomes (24). This could facilitate timely management of later co-morbidities, such as growth failure, cardiac and metabolic conditions, and pubertal and fertility problems.

### 2.3 45,X/46,XY mosaicism

This karyotype has been reported to occur in 1 in 15,000 live births (29), although this is likely an underestimation. Individuals who have 45,X/46,XY mosaicism, including those with structural defects of the Y chromosome, can have phenotypically male, female or atypical genitalia, depending on the degree of embryonic “testicularization” of the gonads and subsequent intra-uterine testosterone production (30–32). Only girls with this karyotype, and having one or more typical clinical manifestations, are referred to as having Turner syndrome (1).

There have been few case series of phenotypic characteristics and outcomes of individuals who have 45,X/46,XY mosaicism. Boys with this karyotype, similar to girls who have Turner syndrome, are generally reported to be short-statured, although this may not be evident until after 2 years of age (33), and to have varying degrees of fertility problems (31, 34–36). Diagnosis may occur only in adulthood as part of the work-up due to fertility problems (34, 36, 37). Karyotype assessment should be considered in boys with unexplained short stature, particularly in the presence of genital anomalies and/or phenotypic features suggestive of Turner syndrome (e.g. short 4^th^ metacarpal/metatarsal, webbing of the neck, lymphedema). However, reports of such features are biased towards patients who receive a postnatal diagnosis and karyotype analysis due to growth or fertility issues (35); there is very limited evidence of growth disorder, fertility problems or abnormal gonadal histology in individuals who are otherwise asymptomatic. Prenatal assessment, using non-invasive diagnostic techniques, may provide less biased information (18, 31, 34). Prenatal counseling of future parents who learn that their child has a 45,X/46,XY karyotype is not straightforward. Approximately 90-95% of cases who have a prenatal diagnosis are reported to be phenotypical males, whereas the proportion is much lower and with more variable phenotype in individuals who are diagnosed postnatally (31, 35).

### 2.4 Klinefelter syndrome and variants

The diagnosis of individuals who have Klinefelter syndrome and variants of 47,XXY, such as mosaic forms or multiple sex chromosome aneuploidy, remains challenging, with many patients misdiagnosed, undiagnosed or only diagnosed as adults (38). Reported prevalence varies widely from 85 to 250 per 100,000 males, but depends greatly on time of diagnosis; reported prevalence for postnatal diagnosis is very much lower than that for prenatal diagnosis (38, 39). While the observed prevalence for postnatal diagnoses has been increasing over time, up to 75% may remain undiagnosed (27, 38). Including cases diagnosed prenatally or at birth, the median age of diagnosis is around 27 years and most frequently due to examination for fertility problems in adulthood. Due to few clinical signs prior to puberty, only about 10% of expected cases are diagnosed during childhood (2). Prenatal diagnosis due to invasive screening was usually incidental until recently, when non-invasive screening was introduced. Similar to Turner syndrome, standardized neonatal genetic screening is now possible (2, 40) and would identify the true prevalence more accurately, enabling improved early management of co-morbidities (2, 38). Prospective studies are needed to establish the cost-benefit ratio of such nationwide/regional screening initiatives, because young boys who have Klinefelter syndrome are generally reported to experience few clinical problems. However, a new study suggests that even undiagnosed males with Klinefelter syndrome show many, if not all, of the phenotypic characteristics normally attributed to the syndrome (41).

The clinical phenotype is variable and the relationships with genetic mechanisms are not well understood (42). Gene dosage effects and chromosome inactivation may be involved, but impaired protein-protein interactions may also play a role (43). Furthermore, alterations in DNA methylation have been identified that may affect imprinted genes through epigenetic mechanisms (38, 42, 44), and alterations in the genetic regulatory machinery, such as circular RNA and micro-RNA, may be found across multiple tissues of patients with Klinefelter syndrome and Turner syndrome (45). These different genetic factors and mechanisms complicate understanding of the genotype-phenotype correlations. Testicular degeneration is observed in the majority of young men who have Klinefelter syndrome, as a consequence of germ cells loss initiated during early development, in combination with disturbed maturation of Sertoli and Leydig cells (46).

## 3 Follow-up of patients with sex chromosome variations

### 3.1 Stature

One of the most frequent characteristics of girls who have Turner syndrome or 45,X/46,XY mosaicism is short stature. This may be a clue to the diagnosis, but comparison with familial target height may be needed if the parents are tall. The cause is partly due to *SHOX* (short stature homeobox) gene haploinsufficiency, which explains some of the skeletal anomalies and some of the reduced height (1, 47, 48). Growth hormone (GH) is a well-established approved therapy for growth failure in girls who have Turner syndrome (1, 49). To enable a longer period of therapy, it is recommended to start GH early, ideally at 4-6 years of age, which requires early testing and is not possible when diagnosis is delayed. Meta-analysis of published data showed an increase in adult height for girls who have Turner syndrome treated with GH, irrespective of karyotype, and this was further increased when oxandrolone was added (50). Adult height within target range is more likely when GH is given (51), but adult height gain is reduced if there is interruption of GH therapy (52) and if GH dose is titrated according to insulin-like growth factor-1 level (53). In order to provide optimal height outcome, the dose should be appropriate for the indication; supraphysiological IGF-1 levels (between +2 and +3 SDS) can be tolerated according to clinical judgement, and adherence with therapy should be maintained (1, 54).

Several reports have shown deficient growth in boys who have 45,X/46,XY mosaicism, with growth deceleration from about 2 years and a poor pubertal growth spurt, although puberty generally occurs spontaneously (33–36). Both boys and girls with 45,X/46,XY mosaicism may have reduced GH secretion or GH insensitivity, possibly due to *SHOX* haploinsufficiency (47). GH therapy can improve short-term growth in boys with mosaicism (31, 35, 55, 56). Although there is a gain in adult height standard deviation score, adult height has not been shown to be significantly greater with GH therapy than without and more detailed long-term studies are needed to establish the effectiveness of GH therapy (36, 55, 56). Genotype-phenotype correlations, including features related to growth outcomes, are extremely difficult to establish in individuals who have mosaic NSVSC (56). Reasons may include rareness of the individual variations, tissue heterogeneity with regard to degrees of mosaicism, and inconsistent phenotypic descriptions.

Males who have Klinefelter syndrome, 47,XXY and variants, generally grow taller than expected (approximately 1 SD above genetic height potential at adult height), probably due to the extra *SHOX* gene and delay in epiphyseal fusion caused by testosterone deficiency (2, 38, 48). Longitudinal growth is usually unremarkable in infancy and early childhood, increasing from about 2-6 years of age, with increased leg length (57, 58), and associated with increased central adiposity, decreased lean body mass and an elevated risk of developing metabolic syndrome and diabetes mellitus (2, 38). Testosterone levels are generally reported to be low normal or slightly below the age-specific reference in adolescent boys who have Klinefelter syndrome, and replacement therapy has been shown to partly compensate for the body composition and metabolic disorders (2, 58–60).

### 3.2 Co-morbidities

Care for individuals who have a NSVSC should be interdisciplinary, lifelong, transparent, and embedded in a stepwise process of shared decision-making, in which the child is involved as early as possible in an age-appropriate fashion. A key person is the child psychologist, who can provide psychosocial support and therapy, and oversee a shared decision-making process (61). Other specialists will vary according to the karyotype, the severity of the condition and the age of the patient, but most often include a pediatric endocrinologist, a pediatric urologist (in case of atypical-appearance genitalia) and a gynecologist. Surgical interventions to make the genital aspect more typical-looking and without any expected functional improvement resulting from the surgery should only be performed on active request of the child concerned (4, 61). As the child gets older, an endocrinologist, a fertility specialist and a sexologist will likely become important. Few teams have clinical specialists (e.g. patient navigator, social worker) to facilitate communication with the patient’s micro-environment and to enhance coping. The role of support groups and qualitative online information is vital, and patients and their families need to be actively encouraged to liaise with such groups (4, 61, 62).

A European study of patients aged ≥16 years who had confirmed DSD, including more than half who had numerical or structural variations of the sex chromosomes, reported that 91% had good/very good general health, although it was poorer compared with controls (6). Co-morbidities were present in 94% of women who had Turner syndrome, 87% of men who had Klinefelter syndrome and 82% of those who had 45,X/46,XY mosaicism, significantly greater than controls in each group. Turner syndrome is associated with increased risk of hypertension, cardiovascular disease, metabolic disorders, such as hypothyroidism and diabetes, and hepatic and gastrointestinal disorders (1, 63, 64). Early diagnosis is important for adequate screening for these co-morbidities and effective treatment when present. Management of Turner syndrome requires active and specialized involvement from disciplines such as cardiology, ear/nose/throat, ophthalmology and orthopedics (4, 24).

There is increasing evidence that boys who have a 45,X/46,XY karyotype may have similar and equally frequent features and co-morbidities as girls who have this karyotype, including cardiac, ear/nose/throat and renal problems, and dysmorphic features (30, 31, 34, 56). Boys and girls with this karyotype have increased risk of gonadal germ cell cancer, requiring appropriate gonadal management on a case-by-case basis (33, 35, 65, 66). One risk factor related to development of gonadal germ cell cancer is the presence of the testis specific protein on the Y chromosome (*TSPY*) gene, located on Yp (66). While there are no specific studies, it can be hypothesized that individuals who have structural variations of the Y chromosome, e.g. those who have a 45,X/46Xi(Yp) karyotype and thus an extra copy of *TSPY*, have a risk at least equal to individuals who have the classic 45,X/46,XY variant. Because of the risk of poor growth and other potentially life-threatening conditions, continued follow-up of boys with a 45,X/46,XY karyotype is required, irrespective of the genital appearance at birth (31, 34, 56). Suggested guidelines, based on data from small case series, propose that patients should be followed with regular checks of growth rate, pubertal status, cardiac, ear/nose/throat, autoimmune and metabolic conditions, testicular function and tumor screen (34).

The most frequently observed clinical features in patients who have Klinefelter syndrome are issues associated with infertility, neurocognitive dysfunction with learning disabilities in childhood, increased stature, eunuchoid body proportions and gynecomastia (2, 57). Abdominal adiposity, decreased muscle mass and metabolic syndrome are reported in up to half of Klinefelter syndrome cases, with increased risk for diabetes, cardiovascular disease and thrombotic events (38, 58, 67). There is also a significantly increased risk of morbidity involving many body systems and mortality from various causes (38, 59). Testosterone therapy non-significantly decreases this risk, and further long-term studies are required to determine the place and added value of testosterone therapy in this regard (68). There is a physiological increase in testosterone concentration in male infants at 1 to 4 months of age, the so-called mini-puberty (69). It has been suggested that testosterone therapy during this short period could improve testicular histology and some neurodevelopmental problems, although this remains highly experimental (70). Published guidelines from the European Reference Network on Rare Endocrine Conditions and from the European Association of Andrology recommend that testosterone replacement should not be given before puberty (2, 71). Additional support in school may also be needed due to neurocognitive challenges including the potential for dyslexia (38). Follow-up of patients through adolescence and adulthood is necessary, due to the diversity of the phenotypic features and the morbidity and mortality risks.

## 4 Fertility preservation

### 4.1 Women who have Turner syndrome, including sex chromosome mosaicism

Women who have Turner syndrome experience primary or secondary amenorrhea, premature follicular depletion and ovarian insufficiency that often occurs pre-pubertally (1, 8). While follicle formation is probably normal during early fetal life, at 15 weeks’ gestation apoptosis was seen in nearly 50% of oocytes in 45,X fetuses compared with 3-7% in fetuses with 46,XX karyotype (72, 73). Spontaneous menarche and pregnancy is rare in women who have 45,X karyotype, although less so in those with 45,X/46,XX mosaicism (1, 74, 75). There are also increased risks of pregnancy loss, preterm delivery and neonatal complications following spontaneous pregnancy in women with Turner syndrome (1, 74–77). Cardiac complications in women with congenital cardiac anomalies and risk of aortic aneurysm may further complicate pregnancies (75). Women who have Turner syndrome due to 45,X/46,XY mosaicism are at increased risk of gonadal malignancies, and viable follicles are almost always absent, even in early childhood. Therefore, gonadectomy in childhood is recommended for girls who have this karyotype (1, 78, 79).

Because of the rapid decline in oocytes, options to maximize chances for reproductive potential should be considered following a diagnosis of Turner syndrome. Options for assisted reproduction include cryopreservation, oocyte donation or gestational surrogacy. Cryopreservation of oocytes has developed rapidly over the last 10 to 12 years, using cryoprotective agents during vitrification to prevent the formation of ice crystals that can damage proteins in the cells (80). The procedure has been developed mainly in cancer patients (81, 82) and, in this context, live births occur in about a third of cases where the vitrified oocytes have been used (81); however, the probability of childbirth using vitrified oocytes may be lower in women with Turner syndrome, but more data will be needed before this can be determined. Ovarian stimulation with gonadotropins for oocyte cryopreservation is only possible in adolescents and young women who have experienced spontaneous puberty and menarche, which is mainly confined to those with 45,X/46,XX mosaicism (1, 83–86). Timing is very important and levels of anti-müllerian hormone should be monitored once a year; concentrations should be sufficiently high initially, with ovarian stimulation and oocyte retrieval carried out when levels start to decrease (85). Ovarian stimulation and oocyte retrieval is reported to be safe and effective in adult women. However, because there are very few studies in adolescents, and because the procedure is very intensive and burdensome, it has been offered mostly to women >18 years of age (85, 86), although ovarian stimulation and oocyte retrieval are feasible in selected younger individuals, after multidisciplinary counseling. Pre-implantation genetic testing or prenatal testing can be used to identify chromosomal abnormalities in embryos (85–87), although this is subject to ethical concerns (88). Pregnancies with autologous oocytes have higher than normal rates of chromosomal abnormalities, although it is the surrounding granulosa cells in the follicles that are generally reported to be abnormal rather than the oocytes (89, 90). However, miscarriages are more frequent in women with Turner syndrome, suggesting that other factors such as uterine problems may influence this, and more information on live birth rates following oocyte cryopreservation is required (75–77, 85). The first reported live birth using cryopreserved oocytes in a woman who has Turner syndrome, with 45,X/46,XX karyotype, was published recently (91).

Cryopreservation of ovarian tissue has been performed in some countries, but this approach has not been systematically and rigorously tested (85, 92). There is currently very limited evidence of the efficacy of the technique for women who have Turner syndrome, and questions remain concerning the quality of the granulosa cells and follicles within the tissue (89, 90). Although the technique remains experimental, ovarian tissue cryopreservation in young girls who have Turner syndrome could be feasible if sufficient numbers of follicles are present (93). A protocol for a large observational trial has been published, with the aim to examine pregnancy rates and outcomes (94). Most women who have Turner syndrome and 45,X/46,XY mosaicism have bilateral streak gonads or severe forms of testicular dysgenesis (95, 96). These gonads are mainly removed due to increased risk of gonadal germ cell malignancy and very rarely contain viable oocytes; nonetheless, a template protocol has been proposed for gonadal tissue cryopreservation (97) and a case series that included young female patients who had 45,X/46,XY mosaicism has been published (98). No successful pregnancies have been described so far.

Other options for women who have Turner syndrome include oocyte donation, although this is not allowed in many countries (85), or adoption. Pregnancy presents an increased risk in patients with severe cardiac conditions and, therefore, prior cardiac and aortic assessment is recommended, and such pregnancies need intensive monitoring by a specialized cardiologist (1, 99). An algorithm for decision on which fertility preservation or reproductive option can be chosen has been proposed, but this is subject to change with increasing scientific knowledge and expertise (85, 100). In all options for motherhood for women who have Turner syndrome, an interdisciplinary perspective is required for full evaluation, safe management of pregnancy, psychological counseling and treatment (1, 85, 97, 98, 101). Patients need to have sufficient psychosocial maturity to understand the implications of fertility preservation and tolerate the potential surgical manipulations. Full education and informed consent are required and because some fertility preservation decisions have to be made during childhood, there are multiple ethical considerations for all parties involved (102). There are long-term costs for cryopreservation and the cost-efficiency of the different options are currently questionable.

### 4.2 Men who have 45,X/46,XY mosaicism

Men who have a diagnosis of 45,X/46,XY mosaicism have varying degrees of testicular dysgenesis (31, 34, 103), which may be related to differences in tissue distribution of the mosaicism (104, 105). Spontaneous puberty and normal pubertal progression is reported for many boys with this mosaic karyotype, but testicular function may decline over time (31, 35, 36, 56, 103). Histological examination of spermatogenesis found no germ cells in 50% of post-pubertal boys with the karyotype, arrested spermatogenesis in 25% and spermatids in 25% (31, 65). Semen analysis showed azoospermia for most, in line with other studies that have also noted a high proportion with azoospermia (34, 35, 37, 65).

Potential fertility problems and possible measures for fertility preservation should be discussed at appropriate times with boys who have 45,X/46,XY mosaicism (31, 34). Ideally, when semen samples indicate no or strongly reduced sperm concentrations, testicular biopsies in the context of germ cell cancer surveillance are combined with a tentative testicular sperm cell extraction (TESE) procedure (31, 34, 56, 66). There have been reports of extracted spermatozoa used for intracytoplasmic sperm injection (106, 107), but there are no established protocols for the procedure and no births reported yet for the technique in males with 45,X/46,XY karyotype (31).

### 4.3 Klinefelter syndrome

Testicular histology in men who have Klinefelter syndrome may vary, but commonly includes testicular degeneration with fibrosis and hyalinization of the seminiferous tubules and Leydig cell hyperplasia, and subsequent impaired testicular function (46, 108, 109). Clinically, this results in testosterone deficiency, hypergonadotropic hypogonadism, small testes and infertility (38, 108). In a European multicenter study, less than 1% of 212 men with Klinefelter syndrome had children without assisted reproductive technology; sperm donation was reported by 38 patients and 79% of the men with Klinefelter syndrome said they knew they could not conceive their biological children (110).

Microdissection TESE (mTESE) has become an established technique for retrieval of intratesticular spermatozoa in recent years; age may be a factor in the success rate, although sperm can be recovered in adolescents, early adulthood and even relatively late adulthood (2, 111, 112). However, there is no current evidence that mTESE carried out pre-pubertal or during adolescence is more successful than in early adulthood (109, 112, 113). Analysis of 386 testicular biopsies in men who had Klinefelter syndrome found that spermatogonia were identified in 83% of pre-pubertal cases and could still be detected in 49% of adult patients (114). In 152 of the adult men who had no mature spermatozoa in their biopsies, 24% had immature spermatogonia, which could potentially be used for *in vitro* maturation. Spermatozoa retrieved from TESE, or for a small proportion of men who have spermatozoa in semen, can be cryopreserved for later use, but there are currently few studies, and it is not clear when sperm retrieval and preservation should ideally be carried out (2, 115, 116).

For men who have Klinefelter syndrome, there have been a number of reports of pregnancies and births using spermatozoa from TESE for intracytoplasmic sperm injection (117–120). In each of these reports, some patients used fresh spermatozoa on the day of oocyte retrieval from the mother and some used cryopreserved spermatozoa. There appeared to be little difference in the pregnancy and live birth rates, but a significantly higher fertilization rate for fresh spermatozoa was reported in one study (117). The proportion of men who had Klinefelter syndrome who underwent TESE and fathered their biological children was between 10% and 28%, and it has been reported that the average is about 16% (2).

There remain many ethical questions with regard to fertility preservation in males with Klinefelter syndrome. The median ages of the men at the time of TESE in the studies reporting pregnancy and live births were approximately 30-34 years (117–120). TESE for sperm retrieval in adolescence or younger has been suggested, which requires sufficient psychological maturity to undergo the procedures and to understand the long-term implications (2, 116, 121). There remain questions such as whether mosaicism affects spermatozoa retrieval, since most studies have been in non-mosaic Klinefelter syndrome, and whether genetic anomalies are likely to be passed to the offspring (121). Thus, although fertility management in males with Klinefelter syndrome is developing rapidly, further large studies and clinical and ethical guidance are required, particularly in the pediatric population.

## 5 Conclusions

The care of individuals who have NSVSC has evolved greatly over recent years, but there remains a need for consensus on diagnosis and management. Diagnostic techniques for Turner syndrome are not sufficiently effective, with many girls undiagnosed, and surveillance by family doctors and school nurses should be improved. Neonatal screening based on heel prick tests could possibly be performed in all newborn female babies; however, this has many ethical and financial implications. Genetic testing of all newborns would require a strong evidence base for a cost:benefit advantage for long-term health. In the absence of such evidence, population screening with no presence of phenotypic features may be considered intrusive. Many individuals go through life without a diagnosis or evident clinical problems and identification of a genetic anomaly could be stressful to such individuals and have unwanted healthcare implications. Many boys and girls who have 45,X/46,XY mosaicism have short stature and co-morbidities to the same extent as girls with Turner syndrome. Therefore, they should be enrolled in the same surveillance and treatment protocols as girls who have “classic” Turner syndrome. Many boys with Klinefelter syndrome, 47,XXY and variants, are either only diagnosed as adults, due to fertility problems, or remain undiagnosed. The same ethical and financial considerations as for Turner syndrome apply for newborn screening of boys with Klinefelter syndrome.

Children with unexplained short stature should be screened for karyotype to identify girls with Turner syndrome or boys with 45,X/46,XY mosaicism, particularly in the presence of characteristic phenotypic features or atypical genital appearance. GH is an approved therapy for girls who have Turner syndrome and short stature and has been shown to increase adult height. Boys who have 45,X/46,XY mosaicism may have growth deceleration in childhood and a poor pubertal growth spurt. GH therapy can improve their growth, but more long-term studies are needed to establish effectiveness on adult height. In contrast, men who have Klinefelter syndrome tend to grow taller than genetic potential, by about 2-5 cm, partly due to testosterone deficiency causing delayed epiphyseal fusion. The associated reduced lean body mass, increased adiposity and elevated risks of metabolic syndrome, thrombotic events and mortality, may be compensated in part by testosterone therapy, but further studies are required to determine potential efficacy.

A large majority of individuals who have Klinefelter syndrome, Turner syndrome or 45,X/46,XY mosaicism have co-morbidities. Adequate screening and provision of effective treatments for co-morbidities are important throughout life. Care for individuals who have a DSD resulting from NSVSC is complex and should be holistic and personalized. Such care should be centralized in expert centers, and there should be increased awareness that many traditional approaches, including genital surgery, have been abandoned and replaced by a renewed focus on information, psychosocial support and shared decision-making. Close cooperation within an interdisciplinary team, involving patients, parents and patient support groups, is essential to improve management and outcomes.

Fertility potential should be assessed on an individual basis, and discussed with the patient at the earliest opportunity, and in an age-appropriate manner. Oocyte or ovarian tissue cryopreservation is possible in some young individuals who have Turner syndrome. There is an increased risk of miscarriage, preterm delivery or neonatal complications; however, pregnancies and live birth have been reported for a small number of women. Virtually all women who have Turner syndrome with 45,X/46,XY karyotype have no functional gonads and an increased risk for gonadal malignancy; therefore, gonadectomy may be recommended in childhood. Most men with a 45,X/46,XY mosaic karyotype present with azoospermia, but in a small proportion of men, TESE may result in identification of few motile sperm, which theoretically can be used in subsequent assisted reproduction techniques. However, none have currently been reported to result in fathering of children. Many men with Klinefelter syndrome may be able to father a child nowadays using assisted reproduction techniques; studies of TESE in men with Klinefelter syndrome have been reported extensively, with multiple reports of healthy live births worldwide. Optimal healthcare for children born with NSVSC and their parents, requires a multidisciplinary DSD team who can understand and address the ethical questions of fertility preservation in pediatric patients, and international studies and guidelines are urgently needed.

## Author contributions

All authors listed have made a substantial, direct, and intellectual contribution to the work, and approved it for publication.
